# Selection of Methods of Surface Texture Characterisation for Reduction of the Frequency-Based Errors in the Measurement and Data Analysis Processes

**DOI:** 10.3390/s22030791

**Published:** 2022-01-20

**Authors:** Przemysław Podulka

**Affiliations:** Faculty of Mechanical Engineering and Aeronautics, Rzeszow University of Technology, Powstancow Warszawy 8 Street, 35-959 Rzeszow, Poland; p.podulka@prz.edu.pl; Tel.: +48-17-743-2537

**Keywords:** surface texture, measurement, measurement error, measurement noise, profile characterisation, areal analysis, feature characterisation

## Abstract

Processes of surface texture characterisation can be roughly divided into measurement issues and analysis of the results obtained. Both actions can be fraught with various errors, some of which can be analysed with frequency performance. In this paper, various types of surface topographies were studied, e.g., cylinder liners after the plateau-honing process, plateau-honed liners with additionally burnished dimples of various sizes (width and depth), turned, milled, ground, laser-textured, ceramic, composite and some general isotropic topographies, respectively. They were measured with a stylus or via optical (white light interferometry) methods. They were analysed with frequency-based methods, proposed in often applied measuring equipment, e.g., power spectral density, autocorrelation function and spectral analysis. All of the methods were supported by regular (commonly used) algorithms, or filters with (robust) Gaussian, median, spline or Fast Fourier Transform performance, respectively. The main purpose of the paper was to use regular techniques for the improvement of detection and reduction processes regarding the influence of high-frequency noise on the results of surface texture measurements. It was found that for selected types of surface textures, profile (2D) analysis gave more confidential results than areal (3D) characterisation. It was therefore suggested to detect and remove frequency-defined errors with a multi-threaded performance application. In the end, some guidance on how to use regular methods in the analysis of selected types of surface topographies following the reduction of both measurement (high-frequency noise) and data analysis errors was required.

## 1. Introduction

Surface texture can be classified as a fundamental issue for characterising the properties of the manufactured parts that can support the process of control. Much valuable information can be derived directly from the surface texture and the analysis of its value parameters, such as wear-resistance [1], sealing [2], friction [3], lubricant retention [4], tribological (material contact) performance [5], fatigue [6,7], corrosion [8], material properties in general [9]. Moreover, for eco-friendly performance and to reduce fuel consumption and CO_2_ emissions, special, eco-friendly [10] coatings can also be applied.

The whole process of surface texture evaluation [11], including the measurement and analysis of the results obtained, can be fraught with many issues that influence the accuracy of the studies performed. All of those errors can be roughly divided into those typical to the measuring method [12] and those caused by the digitisation or data processing [13], software [14], measuring object [15] or other errors [16,17]. One of the types of errors is facilitated when the measurement process occurs. The errors that occur while the measurement process takes place are often defined as noise [18]. From all of the studied types of measurement noises, e.g., instrument or instrument white [19], random [20], phase [21], signal-to-ratio [22] or, simply, the measurement noise [23], the high-frequency domain (high-frequency measurement noise [24]) is most commonly studied in recent research.

The noise could be generated by aperiodic stochastic vibration caused by random factors during the cutting process and distributed throughout the entire frequency domain. At present, the use of sensors is more robust to environmental issues, but the electrical noise in the sensor output can cause high-frequency noise [25]. Reduction of this type of noise can provide a better resolution, which also reduces the bandwidth of the sensor. In the non-contact, optical measurement of surface texture, according to the non-uniform distribution of the light intensity, the extracted signal is liable to be skewed and asymmetric and can contain a lot of high-frequency noise. The high-frequency noise can be caused by the instability of the mechanics with any influences from the environment or by internal electrical noise. Nevertheless, the high-frequency noise, in most cases, is the result of vibration [26].

Generally, raw measured data, studied as a signal, are composed of the background signal, which is characterised by low frequency, noise signal of high frequency and the useful signal, which needs to be processed separately. The analysis of relevant elements of surface texture, when noise and background data are excluded, can provide a piece of valuable information on how the manufacturing process has proceeded. According to the ISO standards [27], the form (F-operator) or noise (S-operator) should be separated correctly. Increasing errors in the definition of the S–F surface (results of removing the F-surface and S-surface from raw measured data), as a surface after application of F- and S- operators, can cause the wrong classification of properly constructed parts, ultimately causing their rejection [28]. Therefore, a proper selection of methods for the definition of an S–F surface is required. The application of regular procedures, e.g., those commonly available in the software equipment, require, mindful users [29]. The separation of irrelevant components (F- and S-components in the process of S–F surface definition) from the results of surface texture measurements is required so they can be evaluated with frequency-based methods and procedures.

From all of the items mentioned, valuable information on how to use the regularly (available, i.a., in the commercial software) methods is still rare. Many papers consider only brief suggestions about errors that can be received when those methods are applied inaccurately. Most studies concentrate more on the selection of methods instead of the reduction of errors in the data processing procedure. Many papers proposed new, more sophisticated algorithms and procedures rather than providing the reader with simple directions on how to use regular techniques appropriately. Moreover, the influence of the false application of the commonly-used algorithms on the results obtained was also not comprehensively studied.

Therefore, from all of the above, in this paper, various frequency-based methods are proposed for the definition (detection) and reduction (minimisation) of high-frequency measurement errors. All of the actions were supported with regular, commonly available (from the commercial software) algorithms to provide valuable guidance for a regular user. Studies were provided with a profile and areal performance so that both types of analysis (2D and 3D), with specifications of values of surface texture parameters, can be increasingly valuable in many engineering appliances [30].

## 2. Materials and Methods

### 2.1. Analysed Details

Many types of surface topographies were studied such as: cylinder liners after the plateau-honing process, plateau-honed liners with additionally burnished dimples of various sizes (width ranging from 0.07 to 0.8 mm and depth between 7 and 100 µm), turned, milled, ground, laser-textured (with 60- or 120-angle texturing), ceramic, composites and some isotropic topographies in general.

All the studied surfaces were provided, as a preliminary analysis, with an areal form removal process. This digital action, was presented in detail in a previous paper, published by the author; therefore, it was not studied in the current research as many detailed analyses were provided in previously [31] with the application of various degrees of polynomials. During data processing, the received (studied) details were generally flat; they did not contain a form (shape and waviness). Furthermore, all the details (surfaces) were carefully analysed to detect the individual peak (spike) errors from the raw measured data. If surfaces contained this type of peak, they were thresholded; this process was described in detail in [32].

More than 10 surfaces from each type of topography were studied (measured), but only some of them, consistent with general observations to all analysed (presented) data (results), were presented in detail. Moreover, more than 10 surfaces with modelled high-frequency measurement errors were analysed and compared with those measured.

### 2.2. Measurement Process

Analysed details were measured by various techniques, stylus or optical, for providing general proposals for different measuring equipment. The stylus instrument was a Talyscan 150 with a nominal tip radius of approximately 2 μm and a cone taper angle of 60°. The height resolution was 10 nm, the measured area was 5 × 5 mm with 1000 × 1000 measured points and a sampling interval of 5 μm. The measurement speed was 1 mm/s and, correspondingly, its influence was not the preliminary focus of this research; that issue has been comprehensively studied in previous papers.

The non-contact measurement was completed with the white light interferometer Talysurf CCI Lite. The height resolution was 0.01 nm, the measured area was 3.35 × 3.35 mm with 1024 × 1024 measured points and the spacing was 3.27 μm. The effect of sampling on areal texture parameters was not studied in this paper.

### 2.3. Applied Methods

Studied surfaces were analysed by various methods to reduce the influence of the high-frequency errors on the results of surface texture measurements. It was found in previous studies that the detection of high-frequency measurement errors can be improved with the graph performance of power spectral density (PSD), auto correlation function (ACF) and frequency spectrum (FS).

PSD, in its two-dimensional form, is designated as the preferred means of specifying surface roughness with regards to the international draft drawing standard for surface texture analysis but a method concerning the variance of the PSD estimate reduction should also be proposed [33]. Using a PSD, it is possible to characterise turning with regard to the applied cooling methods. Moreover, it enables the determination of the amplitude of the feed component when cutting tool vibrations and tool edge wear is dominant on surfaces; therefore, the results based on the PSD studies can be useful for the identification of surface damages and simplifying surface quality [34]. When a direct comparison of metrology data was obtained by instruments with different spatial bandwidths, PSD was also performed on texture data from a range of optical surfaces of varying quality and manufacturing techniques [35]. In some particular cases, the PSD enabled the derivation of the surface roughness and thus provided useful information on characteristic features which compose the microstructure of the films [36] or, specifically, for optical thin films [37]. Furthermore, the PSD for the surface topography contained significantly more irregularities with dominant wavelengths than the feed per revolution value. These dependencies have not been observed during turning in stable dynamical conditions [38]. Therefore, generally, surface roughness can be thoroughly evaluated by frequency spectral analysis.

The ACF assessment provides practical advice regarding the autocorrelation length and its properties as a function of surface irregularities [39]. Quoting the PSD and the ACF functions, ACF is more accurate for the study of irregular surfaces and PSD for the analysis of periodic surfaces [40]. Some methods of deducing higher orders of autocorrelation lengths are needed to evaluate scanning probe microscopy (SPM) images with non-random distribution of roughness amplitudes. These characteristic values of the ACF could play a key role in further statistical calculations, e.g., on how surface roughness alters the wetting behaviour of liquid helium adsorbed on the caesium surfaces [41]. Moreover, the rough surface can be generated using a 2D digital filter and the Fourier analysis method with controlled ACF and height distribution [42].

The frequency-based methods for the investigation of the surface properties, especially in the process of evaluating surface roughness parameters, can play an important role in the characterisation of some surface properties, such as those required for eco-friendly performance [43,44]. The formation of surface roughness in ultra-precision diamond turning can be investigated using a multi-spectrum analysis method. Within this method, features on a diamond-turned surface can be extracted and analysed by the spectrum analysis of its surface roughness profiles, measured at a finite number of radial sections of the turned surface [45]. It was also found that FS analysis can be valuable for the frequency-based decomposition (FBDA [46]) of some measurement errors when a turned or ground surface is considered. Therefore, FBDA can give more direct results than commonly used (e.g., those available in commercial software) algorithms when the denoising process is completed. Moreover, in the past, FS and sound pressure levels related to surface texture (surface roughness in particular) have been studied for concentrated contacts, such as a stylus or hemispherical tip pin on a rough surface [47].

Reduction of the high-frequency measurement errors was proposed with a comparison of the commonly available (in the commercial software) algorithms, e.g., regular Gaussian (GF), robust Gaussian (RGF), median (MF), spline (SF) or, fast Fourier transform filters (FFTF). GF t has been widely presented in many standards considering an evaluation of surface roughness. First introduced in ISO 11562 in 1996 [48] and replaced (revised) in ISO 16610-21 in 2011, it is very popular in the analysis of surface texture [49]. However, in surface roughness measurement, if spikes are included in the primary profile, a problem occurs wherein the GF is unable to extract the shape components [50]. To address this problem, the use of an RGF is often proposed [51]. In previous surface texture studies, it was found that the ranges of effective spatial frequency could be extended through MF without destroying the properties of the fractal surface. Generally, median filtering expands the effective spatial frequency, enhances the effective resolution and significantly increases the use of the optical profiler [52]. Very popular in the measurement and analysis of surface texture are splines [53]. The most relevant advantages of this type of filtering method, in contrast to the regular Gaussian approaches, were previously indicated [54]. A two-dimensional isotropic spline filter was proposed, among other algorithms, for the separation of the roughness, waviness and form components of the surface texture. The fractional spline filter, often called the universal spline filter [55], is calculated by a fast Fourier transform. The fast Fourier characterisation (FFTF) of the surface texture was utilised in many previous studies [56] considering an analysis of the various type of surface texture, e.g., honed cylinder liner topographies [57]. In presented studies, various procedures, supported by various commonly available methods, e.g., those for noise identifications (PSD, ACF, FS) or reduction (GF, RGF, MF, SF, FFTF), were proposed for the definition and reduction of high-frequency measurement errors. The influence of its application on the values of surface texture parameters from ISO 25178 standards, were also studied in detail. Conclusions were provided with consideration of both measured and modelled data.

## 3. Results and Discussion

The processes of data analysis were divided into those concerning the detection and the reduction of the influence of high-frequency measurement errors on the values of surface texture parameters. In the first part of Section 3 and Section 3.1, a comparison with utility definition for both profile and areal surface texture characterisation was performed. All of the results provided were improved with modelled data (Section 3.2), and guidance for raw measured data analysis (Section 3.3) was proposed with the application of a commonly-used (available in commercial software for a regular user) analysis method for various surface topographies.

### 3.1. Comparing of Methods of High-Frequency Errors Detection with Application of Profile (2D) and Areal (3D) Characterisation

Processes of detection of high-frequency measurement errors can be approved and varied with profile and areal performance. Even surface texture is analysed with 3D (areal) characterisation. Some digital actions, e.g., the detection of frequency-defined measurement errors, can be improved and give more relevant results when a 2D (profile) analysis is proposed. The subsection or extraction of relevant profiles from raw measured data can be aquired instead of analysing the whole received dataset. The reduction of data processing time can be its advantageou; nevertheless, this was not the main purpose and objective of the study performed.

**Figure 1:**
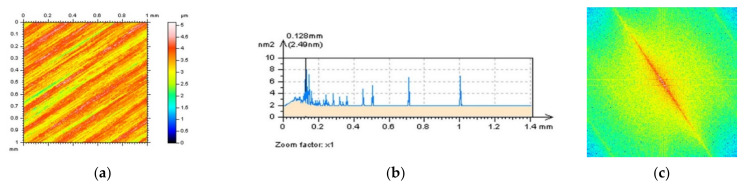

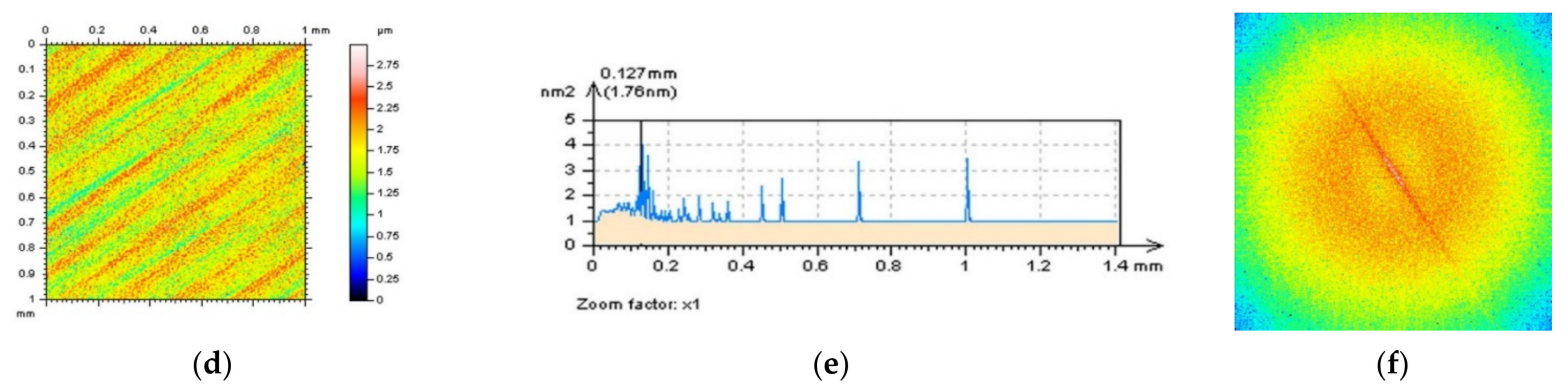
Countour map plots (**a**,**d**), PSDs (**b**,**e**) and FSs (**c**,**f**) were received for optical measurement of milled surface, surface M1(1) without a noise (upper raw) and surface M1(2) containing a high-frequency measurement error (down raw).

**Figure 2:**
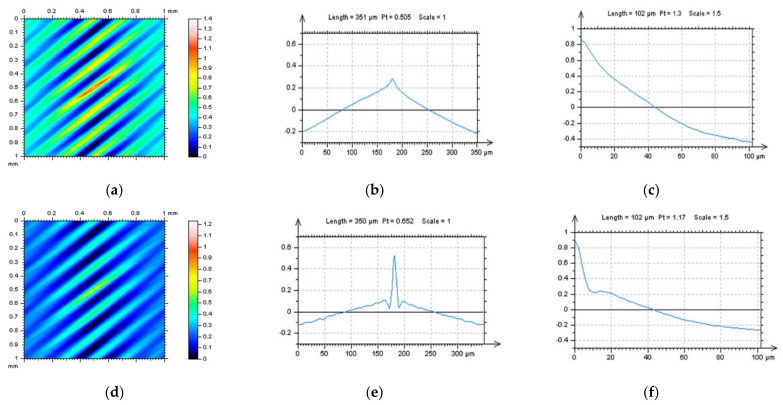
ACFs analysis received for surfaces M1(1) (**a**–**c**) and M1(2) (**d**–**f**), ACFs for surfaces (**a**,**d**), their extracted (**b**,**e**) and zoomed centre (**c**,**f**) profile parts with length equal to (approximately) 400 µm (medium column) and 100 µm (right column).

**Figure 3:**
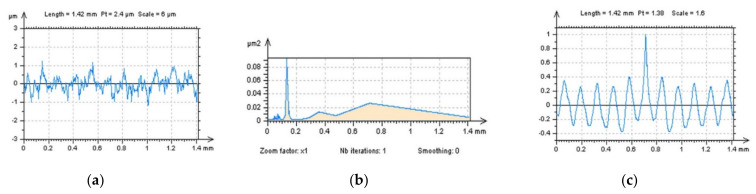

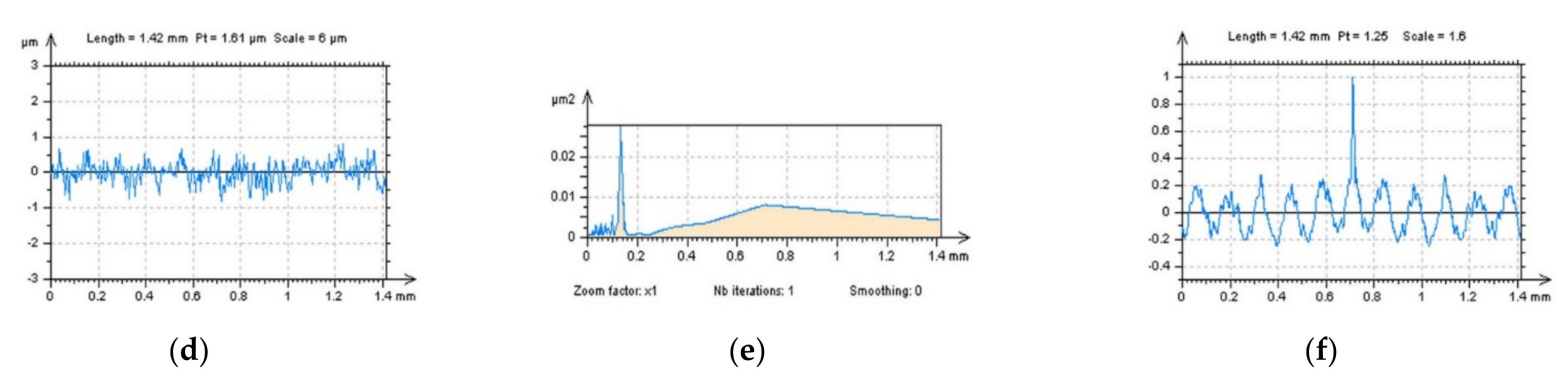
Selected profiles (**a**,**d**), their PSDs (**b**,**e**) and ACFs (**c**,**f**) for surfaces M1(1) (**a**–**c**) and M1(2) (**d**–**f**).

**Figure 4:**
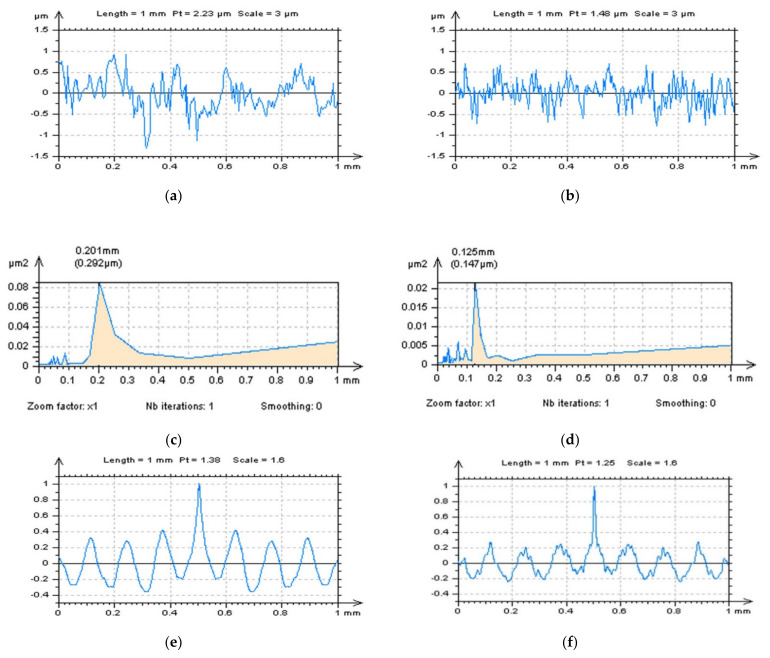

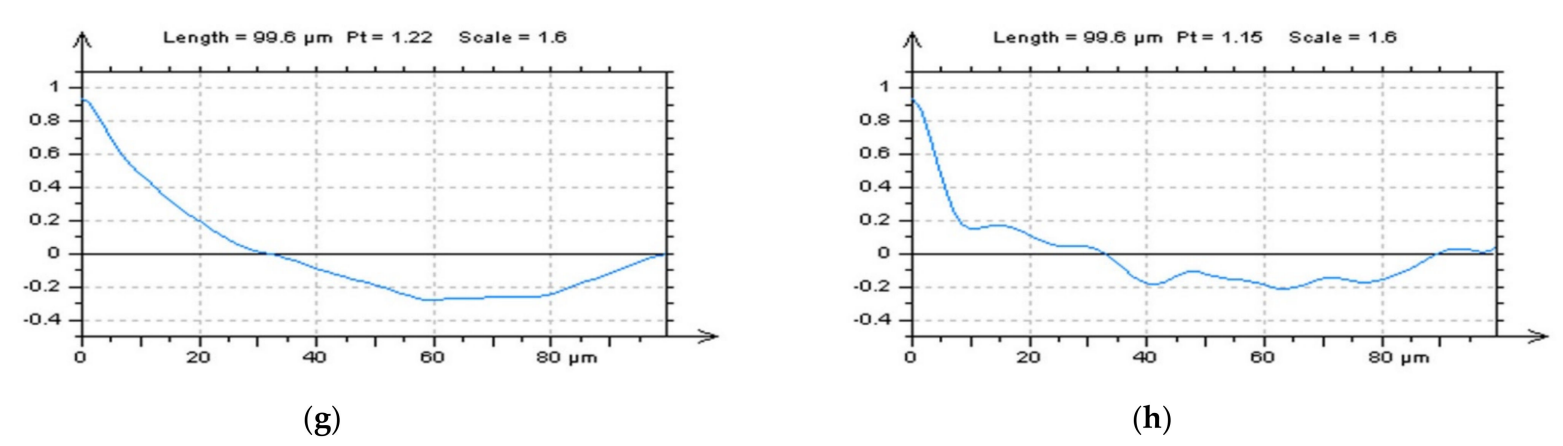
Profile (2D) analysis of the high-frequency measurement errors: measured profiles (**a**,**b**), their PSDs (**c**,**d**) and ACFs (**e**,**f**), and enlarged parts of the ACF graphs centres (**g**,**h**), received from milled surfaces M1(1) (left) and M1(2) (right column), respectively, after application of the TTP technique.

In Figure 1, milled surface topographies were presented with contour map plots (Figure 1a,d), their PSDs (Figure 1b,e) and FSs (Figure 1c,f), respectively. The topographies were measured with an optical method. Considering the analysis of the contour map plots, we can observe differences in the received data; in the second contour map plot (bottom row), noise data can be observed. However, differences in the PSDs can be negligible or, in some cases, did not occur. Some differences could be found for the FS graphs. More visible changes could be found when the ACF graphs (Figure 2) were analysed, especially when the centre-part of the ACF was considered. Therefore, when milled surfaces are studied, it is suggested to analyse PSD and ACF graphs of the selected profiles (2D), in addition to the FS of the raw measured areal (3D) data. Moreover, in some cases, the profile characteristic can not be convincing, especially when vertical or horizontal profiles are considered (Figure 3). In this case, the treatment trace technique, based on the profile characterisation, can be additionally applied. This technique was presented widely in a previous paper by the author, defined as a treatment trace profile (TTP) [26] or, a simplifying, treatment direction method (TDM) [31]. This method can, in this case, improve the profile (2D) utility over an areal (3D) when high-frequency measurement noise is detected. The results of the application of the TTP technique are presented in Figure 4.

**Figure 5:**
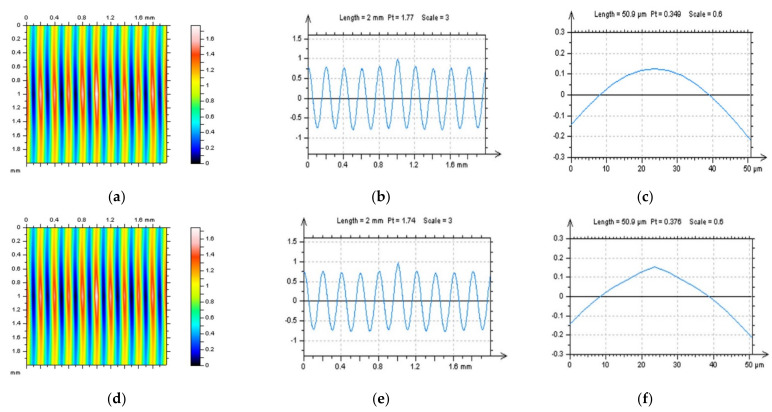
ACFs of turned surface (left column), (middle column) extracted from the middle of the ACF surface graph, of their profiles, and zoomed centries of those functions (right column), received from the measured surface (**a**–**c**) and surface with a high-frequency measurement error (**d**–**f**).

Generally, the TTP method can be valuable in high-frequency noise detection for any anisotropic topographies, especially when a treatment direction can be defined. It was found that turned surfaces can also be directly studied with a proposed method (Figure 5). Nevertheless, high-frequency noise can easily be found via the analysis of the ACF graphs. When the centre of an ACF was studied alongside profile exploration, the amplitude of a value growth increased more rapidly when high-frequency errors were found. This trend has already been noticed before when an autocorrelation function centre-shape method (ACF-CSM) was developed [26]. For plateau-honed cylinder liners, the TTP can be decisive. Nevertheless, if this type of surface texture contains oil pockets (valleys, dimples in general), the valley excluding (extraction) method (VEM [58]) is required.

**Figure 6:**
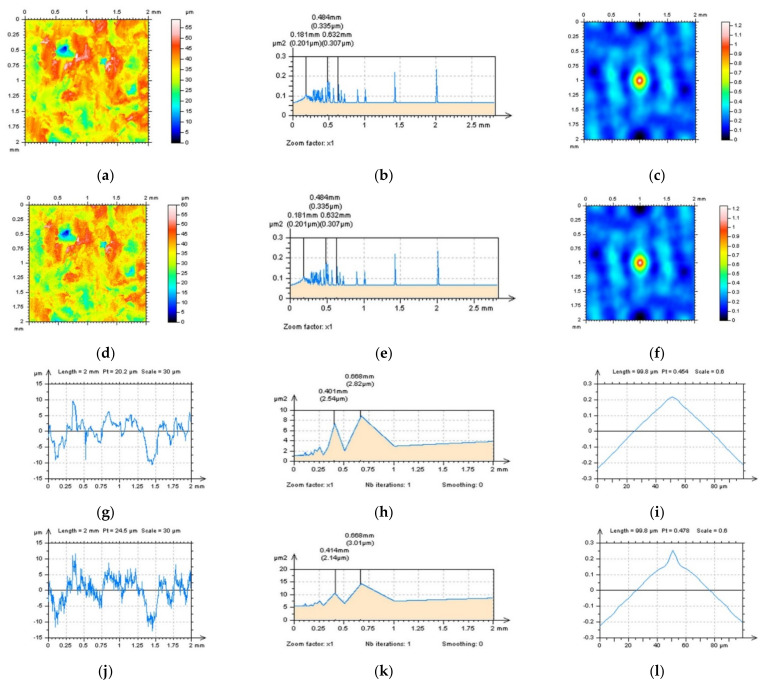
Composite surface texture with raw measured data (**a**–**c**,**g**–**i**) and data containing high-frequency errors (**d**–**f**,**j**–**l**), contour map plots (**a**,**d**), PSDs (**b**,**e**) and ACFs (**c**,**f**) of surface profiles (**g**,**j**) and their PSDs (**h**,**k**) and ACFs (**i**,**l**), respectively.

More complicated is the detection of high-frequency errors when the surface does not contain treatment traces in which direction can be defined. This issue concerns the isotropic surfaces in general. Some examples of this type of surface can be those measured from composite or ceramic machined details (materials). An example of an analysis of a ceramic surface is presented in Figure 6. As in many cases, when considering various types of surface topographies, usually, high-frequency noise can be noticed with an ‘eye-view’ analysis in which differences in contour map plots are easy to follow Figure 6a,d. However, this method can be fraught with many errors that depend on the experience of the analyst and mindful use of the topographic data. Therefore, this method may not be entirely persuasive, even for a user who found it substantially improved in previous, extensive studies. Furthermore, this ‘experience’ technique may not determine how existing errors can be classified and, subsequently, reduced. Analysis of 3D PSDs or ACFs may not give the required response that, as shown in Figure 6b,c,e,f, usually do not exist or, in most cases, are negligible.

When an areal (3D) analysis may not provide some key advantages, a profile characterisation is required. The eye-view studies of the profile Figure 6g,j indicated an occurrence of high-frequency errors. However, comparing measured data and data with high-frequency noise may not be available when the measurement process is not repeated and a vibration of the measurement device is observed. Therefore, studies of profile PSDs and ACFs so must be efficiently performed despite this. Even the PSD graphs may not present some differences, the ACFs, usually can provide some variation, especially when a centre part of the function is fully analysed.

As mentioned before, the ACF-CSM approach may be valuable, nevertheless, as suggested in previous studies, multi-threaded analysis [26] may be required. When applying a PSD analysis, the regular user must be reserved to use an ‘all direction method’ in deriving the 3D (areal) PSD performance. Moreover, both a ‘zoom factor’ and a ‘smoothing’ method must be reduced to a value equal to ‘1’. The smoothing of the PSD function may not provide valuable information when frequencies with a high value are searched, and, simultaneously, the zooming process may also cause a ‘smoothing’ tendency.

**Figure 7:**
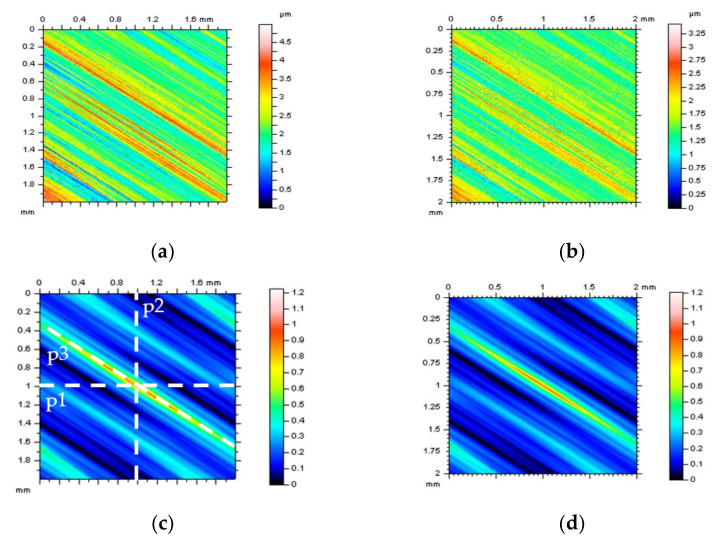
Countour map plots (**a**,**b**) of ground surface texture and their ACFs (**c**,**d**) for raw measured data (left column) and data containing high-frequency errors (right column); in Figure 7c the direction of profile extraction, widely presented in Figure 8, was defined.

**Figure 8:**
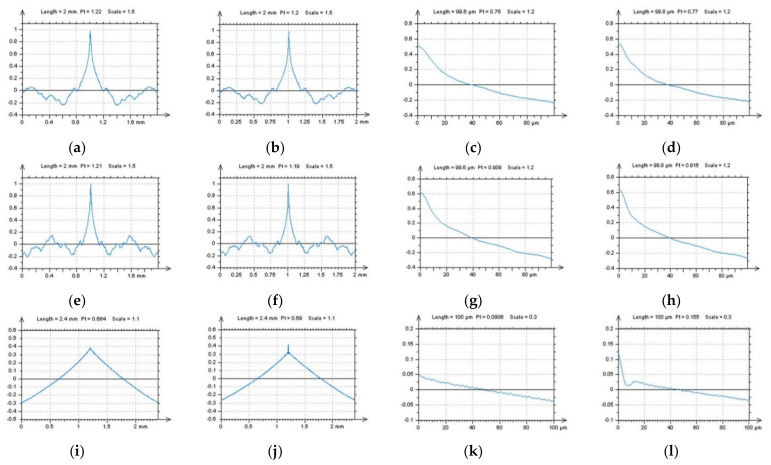
Profiles (2D) of ACF extracted from a 3D (areal) of ACF presented in Figure 7, from raw measured data (**a**,**c**,**e**,**g**,**i**,**k**) and data with high-frequency noise (**b**,**d**,**f**,**h**,**j**,**l**), containing profiles extracted with horizontal p1 (**a**–**d**), vertical p2 (**e**–**h**) and treatment-trace p3 (**i**–**l**) performance (description of the direction of the extraction process was included in Figure 7c).

The ACF graphs can be studied in detail, especially when selected textures with defined directions are observed. In Figure 7, ground surfaces were studied for the detection of high-frequency errors. It was found that differences in the ACF graphs were negligible or did not exist. Similar results were received for profile characteristics when both a horizontal or vertical ACF extraction was proposed. Therefore, respectively, a treatment-trace extraction of an ACF profile was proposed (Figure 7c). Comparing a vertical and horizontal extraction with a treatment-trace approach showed that a high-frequency noise could be defined. Some similar results were obtained when a TTP technique was used for measured data. In Figure 8, differences between vertical, horizontal and, correspondingly, treatment-trace profile (2D) extraction are presented for the analysis of ACF graphs. When the surface contains high-frequency errors, the value of the centre of the ACF profile (Figure 8j,l) increases more rapidly, as was found in previous studies.

### 3.2. Improving Proposed Methods with a Modelled Data

**Figure 9:**
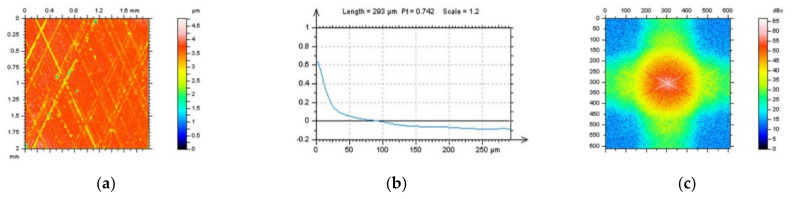

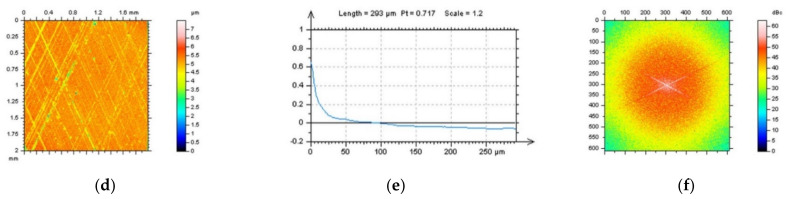
Contour map plots (**a**,**d**), centre-part of a profile ACFs received with a treatment trace technique (**b**,**e**) and FSs (**c**,**f**) from plateau-honed cylinder liner surface with raw measured data (**a**–**c**) and with added a high-frequency noise (**d**–**f**).

For improving presented methods for high-frequency noise detection, raw measured data were added to noise data in the high-frequency domain and with amplitude (Sq) equal to 15% of the amplitude (Sq) of the measured surface. The percentage value was selected according to the previously provided results with high-frequency noise modelling [26]; therefore, it was not considered in this paper. An example of a plateau-honed cylinder liner surface with raw data and the same data with added frequency-based noise were presented in Figure 9a,d, respectively. Some differences could be found when a treatment-trace profile characterisation of an ACF graph was applied (Figure 9b,e). Moreover, some information may also be defined with an FS analysis (Figure 9c,f), in which, differences could be found. Nevertheless, this method was not the main purpose of the studies performed, therefore, it was not comprehensively analysed in this paper.

**Figure 10:**
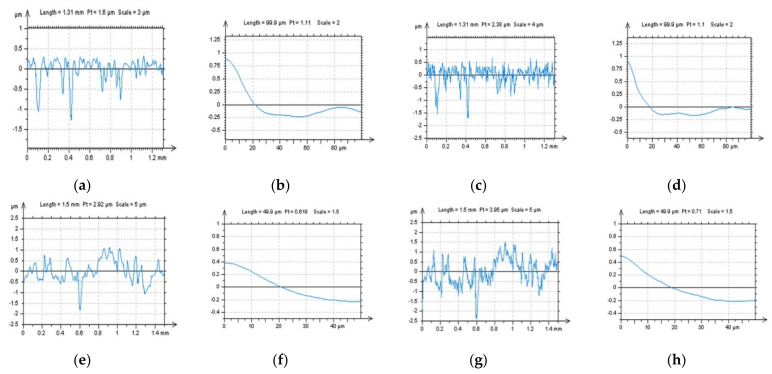

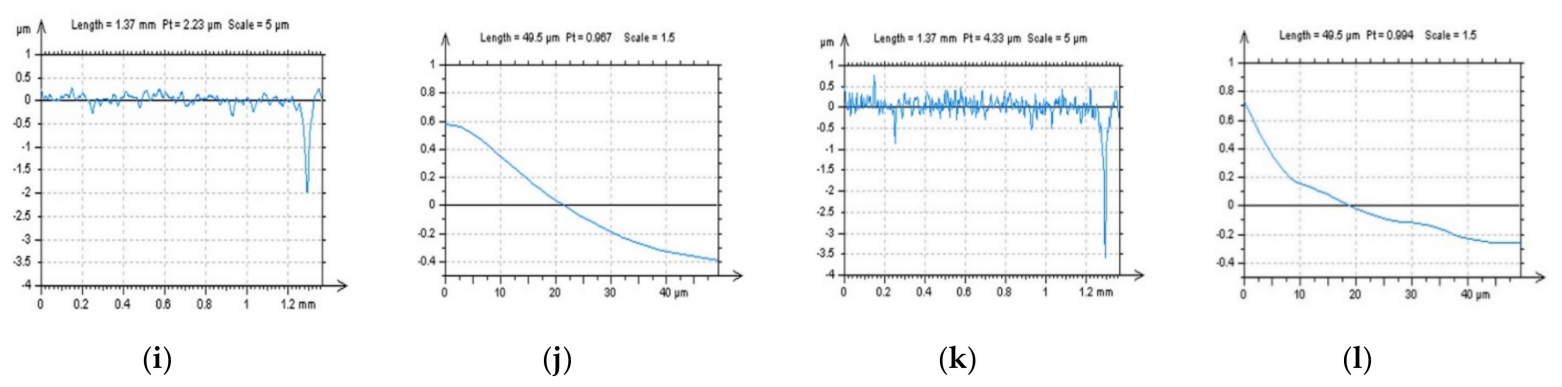
Plateau (**a**–**d**), valley (**e**–**h**) and treatment-trace (**i**–**l**) profiles with their ACFsextracted from a plateau-honed cylinder liner surface presented in Figure 9, received from raw measured data (**a**,**b**,**e**,**f**,**i**,**j**) and data with modelled (added) high-frequency errors (**c**,**d**,**g,h**,**k**,**l**).

The type (location) of the treatment-trace profiles for plateau-honed cylinder surfaces were also studied. An ACF could be studied, in this case, with plateau (profiles selected to the plateau-part of the surface according to the treatment-trace direction), valley (profiles extracted in the valley-part of the surface with a direction related to the treatment-trace direction) or treatment-trace profiles with some plateau and some valley areas of the analysed detail. It was found that the assessment of the PSDs may not allow the reception of an unequivocal response; therefore, detailed studies of the ACF was required, as presented in Figure 10. It was found that the treatment-trace profile characterisation of ACF graphs gave a more relevant response compared to the plateau- or valley- analysis. In some cases, the plateau analysis of the surface could be valuable in high-frequency noise detection. Nevertheless, this method was vulnerable to the existence of the individual peaks or peaks gathered in some studied areas; therefore, for plateau-honed topographies, it is suggested to use a treatment-trace analysis of an ACF rather than plateau characteristics.

One of the main suggestions for this type of surface texture is to select the profiles where its amplitude (height) is relatively small. These types of profiles are those not containing individual peaks, often defined as spikes, deep or wide dimples (e.g., oil pockets, scratches, valleys). If the surface contains many valleys or peaks and it is extremely difficult to select a suitable profile, then it is suggested to find a profile where the density and sizes of the dimples are relatively small. The smallest influence of the dimples (peaks) on the profile height is the biggest accuracy in a high-frequency and noise definition can be found.

**Figure 11:**
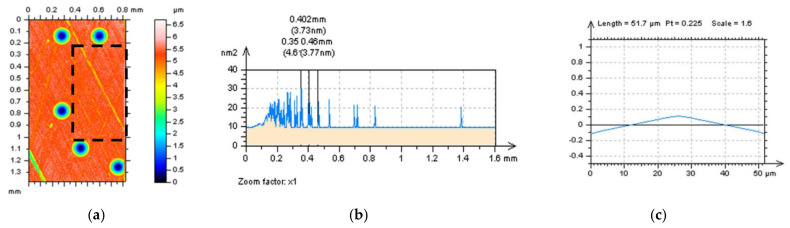

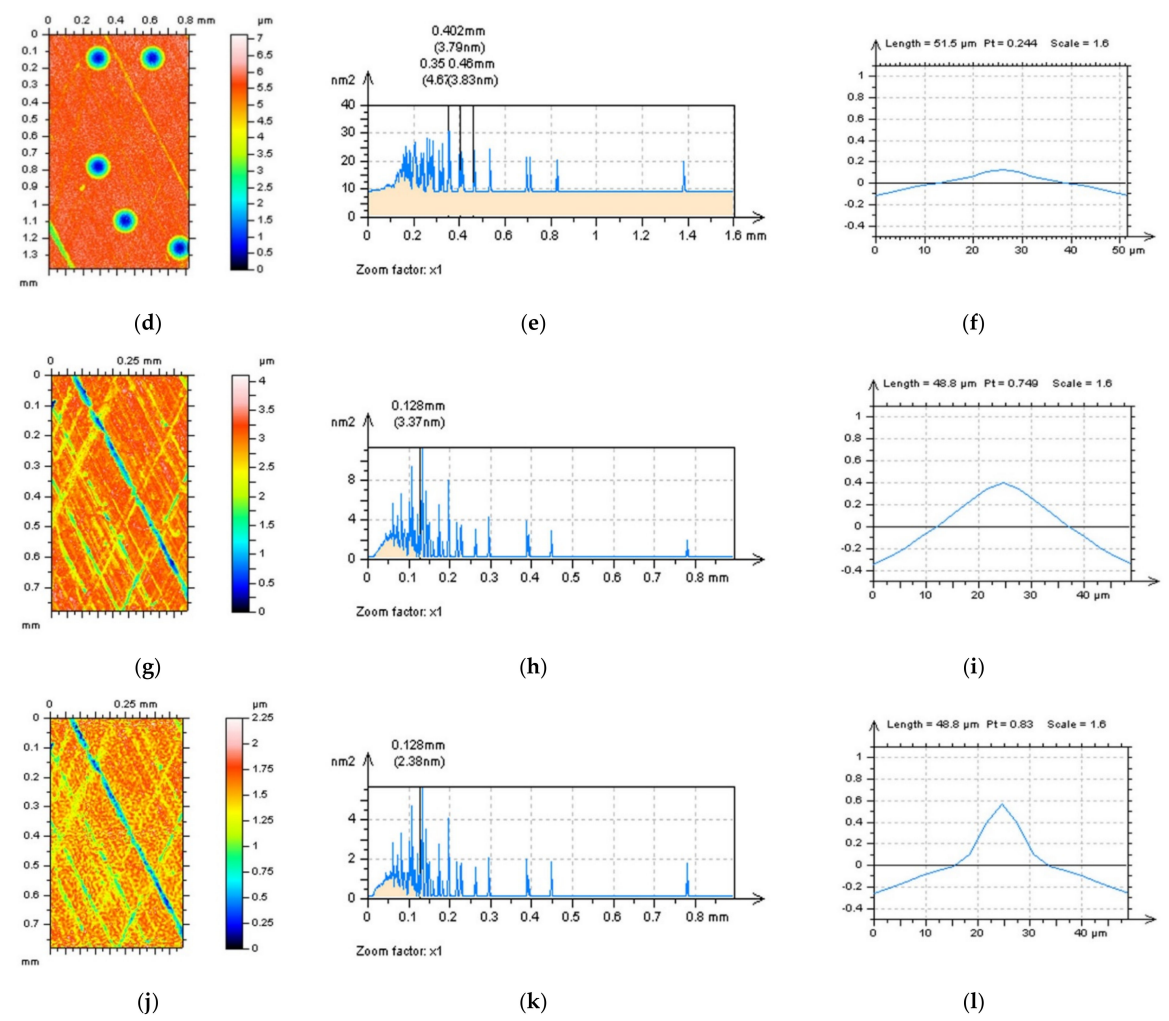
Plateau-honed cylinder liner with additionally burnished dimples (oil pockets) (**a**–**f**) and FOD detail (**g**–**l**) extracted from surface presented in (**d**), contour map plots (**a**,**d**,**g**,**j**), PSDs (**b**,**e**,**h**,**k**) and ACFs (**c**,**f**,**i**,**l**), the analysis proposed for raw measured surface data (**a**–**c**,**g**–**i**) and data with modelled high-frequency errors (**c**–**f**,**j**–**l**).

For plateau-honed surfaces with additionally burnished oil pockets (Figure 11), dimples in general, the occurrence of high-frequency errors were difficult to be defined with both a PSDs and ACFs graphs analysis (Figure 11a–f); however, the changes were, at least, negligible. More accurate methods for high-frequency error definition can be proposed with profile characteristics. One of the proposals can be a 3D analysis of the free of dimple (FOD) areas. This analysis was proposed previously [26,31] when F-filtering methods were provided for an areal form removal of various types of surfaces and was especially encouraging when the results contained wide (and) or deep dimples, wholes, scratches or, subsequently, oil pockets, reducing the distortions in the definition of the reference plane. The FOD approach can be, correspondingly, valuable in the definition of the S–F surface when both operations (F- and S-operation) are applied simultaneously, otherwise, its order affects the results obtained.

The application of a FOD technique was found beneficial for the ACF detection of high-frequency errors; differences were visible for raw measured data and those with modelled noise (Figure 11i,l). Therefore, for surfaces containing valleys, scratches, dimples, oil pockets or, generally, deep (and) or wide treatment traces, it is suggested to analyse details by omitting those features if an areal (3D) characterisation is required.

**Figure 12:**
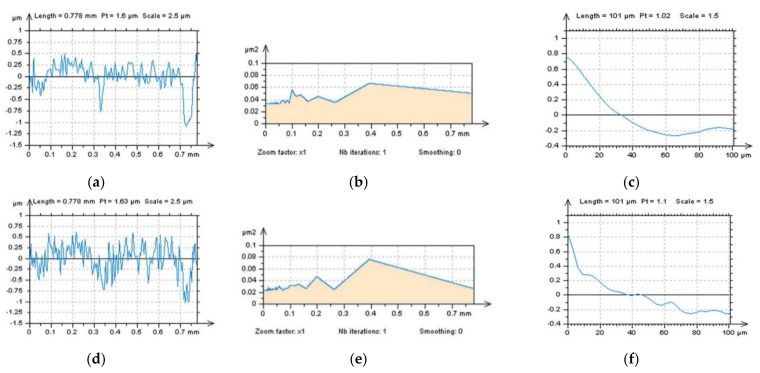
Profiles (**a**,**d**), their PSDs (**b**,**e**) and ACFs (**c**,**f**), respectively, were extracted from the raw measured plateau-honed surface containing oil pockets (**a**–**c**) and the same surface with modelled noise (**d**–**f**).

Detection of high-frequency errors from the results of surface texture measurements of plateau-honed topographies containing oil reservoirs can also be provided with profile studies. In Figure 12, two profiles with raw (Figure 12a–c) and noise-modelled (Figure 12d–f) data were presented, even the differences in PSD graphs were negligible. The centre-parts of ACFs gave more valuable responses regarding high-frequency noise occurrence and the values of ACF increased more rapidly for profiles with noise data.

### 3.3. Proposal of Procedures for Reduction of Frequency-Based Measurement Errors from the Raw Measured Data with Profile (2D) and Areal (3D) Analysis

**Figure 13:**
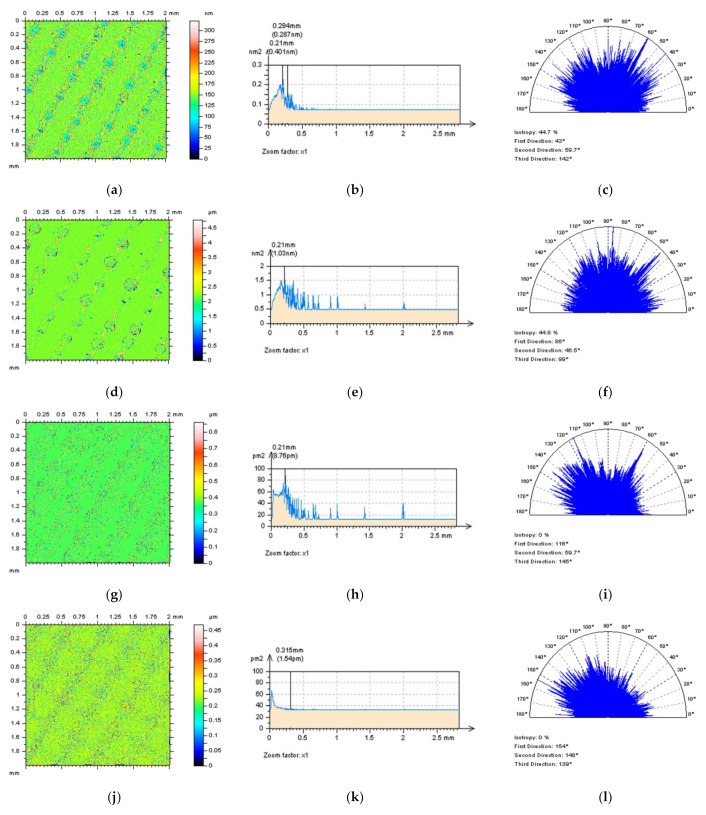

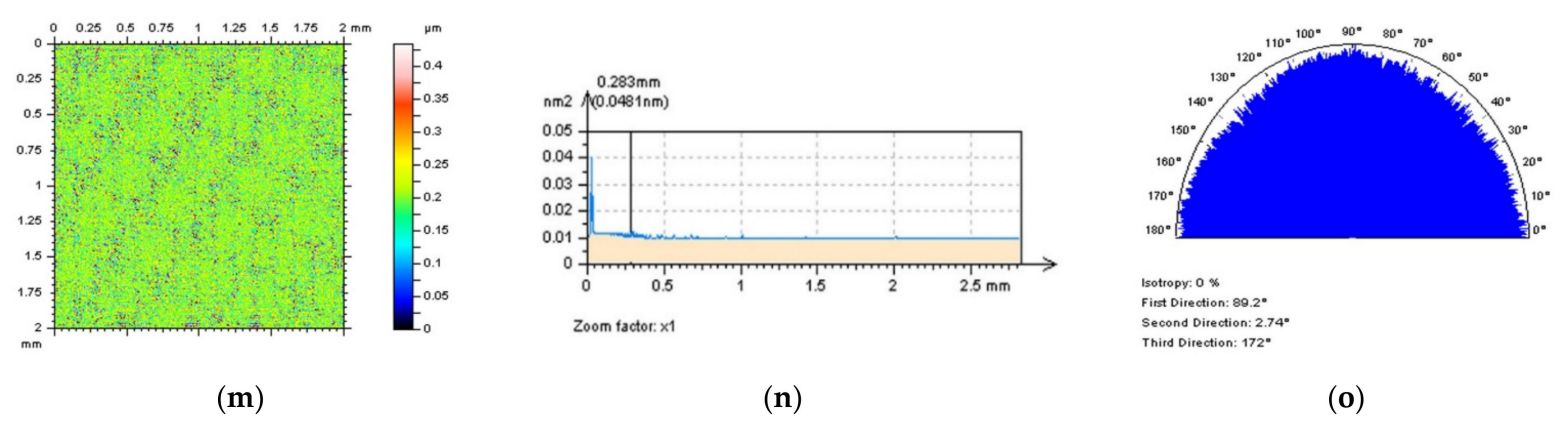
Countour map plots (**a**,**d**,**g**,**j**,**m**), PSDs (**b**,**e**,**h**,**k**,**n**) and texture direction (**c**,**f**,**i**,**l**,**o**) graphs of the HFNS defined by the: Gaussian (**a**–**c**), robust Gaussian (**d**–**f**), median (**g**–**i**), spline (**j**–**l**) and FFTF (**m**–**o**) filter, cut-off = 0.015 mm.

**Figure 14:**
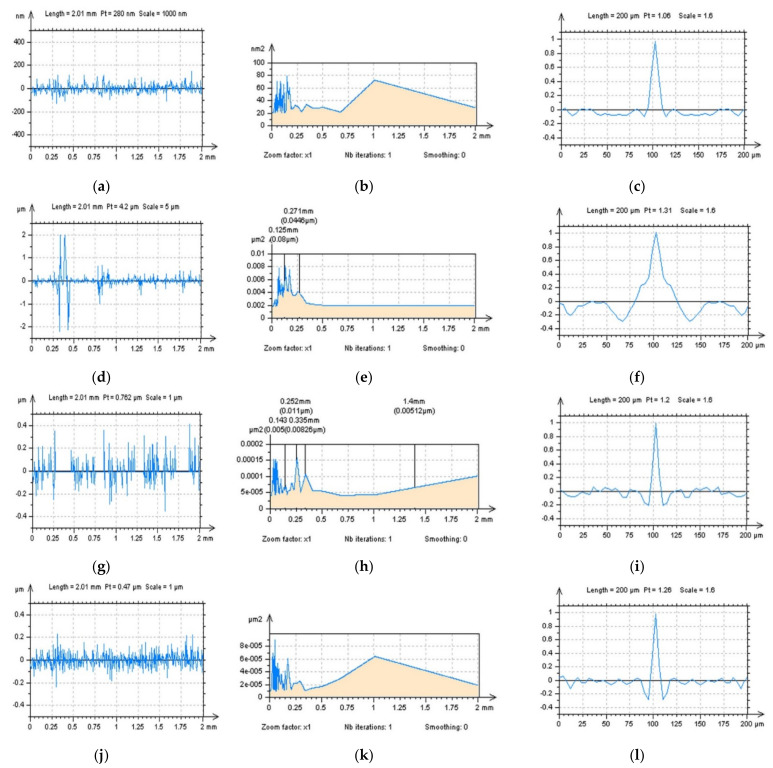

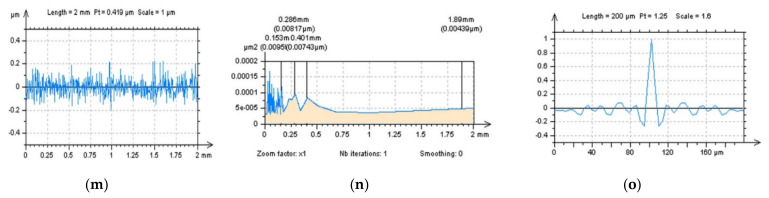
Profiles (left column), their PSDs (middle) and ACFs (right column) of HFNS received by application of Gaussian (**a**–**c**), robust Gaussian (**d**–**f**), median (**g**–**i**), spline (**j**–**l**) and FFTF (**m**–**o**) filter, cut-off = 0.015 mm.

The effect of reducing high-frequency errors can be verified with profile (2D) or areal (3D) performance. In practice, both types of analysis can be applied simultaneously or, in some cases, used separately. One of the proposals of the paper is to analyse the effects of the high-frequency noise removal process. The results of the high-frequency noise separation are defined as ‘high-frequency noise surface’ (HFNS), and its properties are considered key factors in selecting the method of measurement error suppressions. It was defined in previous studies that HFNS should not contain more features than those in the high-frequency domain; therefore, this technique was described as a frequency-based method. When high-frequency errors are removed from the raw measured data, it is expected that HFNS should consist of the high-frequency components or, at least, this frequency should be dominant. These properties can be improved with the analysis of the PSDs and (or) ACFs of the HFNS. High-frequency properties of the HFNS are especially difficult obtain when digital filtering is applied for the characterisation of the non-isotropic surface, e.g., plateau-honed (with oil pockets, dimples, valleys in general as well), turned, ground, milled or laser-textured topographies. Examples of the analysis results of laser-textured surfaces were widely presented in Figure 13 and Figure 14 for areal and profile characterisation, respectively. Three-dimensional (Figure 13) HFNS defined with Gaussian filters (regular or robust) contain other features, in particular, traces of the laser-treatment (a,d). Some traces were visible when a regular median filter was applied (g). Better results were received when isotropic spline (j) or FFTF (m) filters were used. Unwanted features can also be defined via texture direction (right column in Figure 13) graph analysis. Unexpected features (on the HFNS) determine the dominant direction. In practice, the HFNS should be isotropic; therefore any direction is unwanted. From this property, the best results for the laser-texture surface were found when an FFTF method was applied. Analysis of PSDs for both areal (3D) or profile (2D) characterisation gave no suitable response regarding which method is relevant. Moreover, the analysis of the shape of the centre-part of ACFs for the profiles (right column in Figure 14) is not convincing in this case. The nesting index, equal to 0.015 mm, was studied and selected previously [39].

From the proposed techniques, for suppression of the high-frequency errors from the results of surface texture measurements, for isotropic surfaces, both Gaussian, median or spline filters can be suitable. However, special attention must be paid to the selection of the cut-off values; this was not studied in the current paper but was considered in previous studies by the author.

When surfaces have a determined direction or additionally burnished features, such as oil pockets, scratches, dimples or, in general, valleys, the application of the FFTF or regular spline filter gave more relevant results against Gaussian (regular or robust) or median denoising algorithms. From all of the general (commonly-used) methods, those widely available in the commercial software, the FFTF approach seems to be promising.

Differences between each of the PSD, ACF and FS graphs were studied and commented only for the required frequency specifications; high-frequency in particular. These studies can be extended for other frequencies when different types of surface finishing are considered. However, to improve the high-frequency measurement, noise detection and reduction, presented results seem to be sufficiently assured.

Generally, when detecting or removing the high-frequency errors from the raw measured data, it is suggested to observe the PSD, ACF, FS graph for the high-frequency components regarding both areal (3D) and profile (2D) performance. When an areal analysis gives no relevant response, then a profile characterisation is required and, simultaneously, suggested.

## 4. The Outlook

Despite all of the detailed analysis provided, some limitations of the studies can be found. Some of them are planned to be resolved in future prospects. They can be dived into the following categories:

1. Some of the techniques, e.g., those with feature exclusions, may not be useful when worn surfaces are studied. Moreover, the influence of the application of proposed methods on the results of worn textures analysis, including the suppression of the high-frequency noise measurement, will be provided in future studies.
2. The high-frequency noise measurement can be difficult to detect with the proposed methods when the amplitude of errors are relatively small. Moreover, the effect of noise amplitude on the results of applied techniques performance was not studied.
3. It was not determined whether the high-frequency noise can generally gather in some surface areas, such as dimples, wholes, grooves and valleys. Those studies were performed for individual peaks, defined as spikes. Generally, considering digital filtration and the proposed high-frequency noise surface technique, it was assumed that noise was distributed evenly. This must be verified in more sophisticated studies in future.
4. Another limitation can arise with edge-effect minimisation. It was not determined whether boundary conditions influence the accuracy of noise detection and reduction processes. The influence of density and amplitude on errors on the sides (edges) of the measured component was not fully analysed. Moreover, the sharp-edge noise can also be considered for sharp-edged features.
5. The most influencing limitation is that digital filtering can remove important surface details (features). Many suitable studies were performed in current and previous studies (papers) considering an analysis of the removed part of the data, defined as a high-frequency noise surface. This technique requires mindful users as removing essential surface features may completely invalidate the usefulness of the methods proposed.

## 5. Conclusions

From all of the provided results, some concluding, general issues can be raised, as follows:

1. For detection of the high-frequency errors from the results of surface texture measurements, it is suggested to observe the contour map plots, PSDs, ACFs or FSs graphs. In some cases, eye-view analysis of the noise occurrence may not provide a suitable response. Therefore, more sophisticated studies must be performed.
2. When an areal (3D) analysis gave no relevant response regarding an occurrence of high-frequency errors, profile (2D) characterisation was suggested. However, in some cases, the selection of the direction for the extraction of profiles is required. The treatment-trace direction is suitable when a deterministic direction can be defined in analysed detail. Moreover, profiles omitting some deep and wide features, such as oil pockets, scratches, dimples and valleys in general, can be valuable in the process of high-frequency error detection.
3. Except for treatment-trace directions or omitting the valleys, free of dimple analysis can be beneficial in the improvement of a high-frequency errors occurrence. This technique can be especially valuable when plateau-honed topographies with additionally burnished oil pockets are studied. Free of dimples analysis can be beneficial for both the detection and validation of the reduction process of high-frequency-based errors. When profile (2D) characterisation is applied, the process of noise removal should be considered for areal (3D) data in which the contact of materials is areal in general. Moreover, it can be extremely difficult to select a profile that can represent the surface.
4. For the reduction of the high-frequency measurement errors, when isotropic topographies are studied, each of the commonly-used (available in commercial software) algorithms (regular or robust Gaussian, median, spline or Fast Fourier filters) can be valuable and, simultaneously, applied by users. Nevertheless, in some cases, when surfaces contain features in a defined direction (usually the same with the direction of treatment), Fast Fourier Transform or regular isotropic spline filters can give more relevant results for processes of high-frequency errors extractions.
5. It is not suggested to use one value of the nesting index for the S-operation for each type of surface texture; nevertheless, in all of the provided studies, 0.015 mm was applied. Together with supporting the previous studies, it was proposed to analyse the results of a high-frequency measurement noise removal, directly described by the high-frequency noise surface. This type of surface should be characterised by selected distinctive features, described previously with the support of PSD, ACF and FS analysis. All of the mentioned techniques, provided directly by commercial software, can be useful in gaining more confidence in the data received.
6. The main purpose of the presented study was to suggest procedures for selecting appropriate algorithms for the detection and reduction of high-frequency measurement errors directly from the raw surface texture measured data. Therefore, for all of the above issues, it is suggested to analyse PSDs, ACFs and FSs simultaneously. In some cases, one technique may not provide a clear response if the high-frequency errors occur in the raw measured data. For this reason, studies were provided for both measured and modelled data with profile and areal performance, respectively.

## Data Availability

Data sharing is not applicable to this article.

## Nomenclature

The following abbreviations (left) and surface texture parameters (right column) are used in the manuscript:

*ACF* autocorrelation function	*Sa* arithmetic mean height Sa, µm
*ACF-CSM* Autocorrelation Function Centre-Shape Method	*Sal* auto-correlation length, mm
*F-operator* removes F-surface from measured data	*Sbi* surface bearing index
*F-surface* form surface	*Sci* core fluid retention index
*FFTF* Fast Fourier Transform Filter	*Sdq* root mean square gradient
*FOD* free of dimple detail	*Sdr* developed interfacial areal ratio, %
*GF* Gaussian filter	*Sk* core roughness depth, µm
*HFNS* high-frequency noise surface	*Sku* kurtosis
*MF* median filter	*Sp* maximum peak height, µm
*PSD* power spectral density	*Spc* arithmetic mean peak curvature, 1/mm
*PSM* Profile Spline Method	*Spd* peak density, 1/mm^2^
*RGF* robust Gaussian filter	*Spk* reduced summit height, µm
*S–F surface* a surface after S- and F- filtering	*Sq* root mean square height, µm
*S-operator* removes S-surface from measured data	*Ssk* skewness
*S-surface* short-wavelength noise surface	*Std* texture direction, °
*SF* spline filter	*Str* texture parameter
*TTP* treatment trace profile technique	*Sv* maximum valley depth, µm
*VEM* valley excluding method	*Svi* valley fluid retention index
	*Svk* reduced valley depth, µm
	*Sz* the maximum height of surface, µm
